# Beneficial Effects of Polysaccharides on the Epithelial Barrier Function in Intestinal Mucositis

**DOI:** 10.3389/fphys.2021.714846

**Published:** 2021-07-22

**Authors:** Karien Sauruk da Silva, Bruna Carla da Silveira, Laryssa Regis Bueno, Liziane Cristine Malaquias da Silva, Lauany da Silva Fonseca, Elizabeth Soares Fernandes, Daniele Maria-Ferreira

**Affiliations:** ^1^Instituto de Pesquisa Pelé Pequeno Príncipe, Faculdades Pequeno Príncipe, Curitiba, Brazil; ^2^Programa de Pós-graduação em Biotecnologia Aplicada à Saúde da Criança e do Adolescente, Faculdades Pequeno Príncipe, Curitiba, Brazil

**Keywords:** anticancer therapy, intestinal mucositis, natural polysaccharide, mucosal inflammation, intestinal inflammation and injury

## Abstract

Intestinal mucositis is a clinically relevant side effect of anticancer therapies. It is experienced by 60–100% of patients undergoing treatment with high doses of chemotherapy, radiation therapy, and bone marrow transplantation. Intestinal mucositis can manifest as pain, weight loss, inflammation, diarrhea, rectal bleeding, and infection; affecting normal nutritional intake and intestinal function. It often impacts adherence to anticancer therapy as it frequently limits patient’s ability to tolerate treatment, causing schedule delays, interruptions, or premature discontinuation. In some cases, local and systemic secondary infections are observed, increasing the costs toward medical care and hospitalization. Several strategies for managing mucositis are available which do not always halt this condition. In this context, new therapeutic strategies are under investigation to prevent or treat intestinal mucositis. Polysaccharides from natural resources have recently become promising molecules against intestinal damage due to their ability to promote mucosal healing and their anti-inflammatory actions. These effects are associated with the protection of intestinal mucosa and regulation of microbiota and immune system. This review aims to discuss the recent advances of polysaccharides from natural resources as potential therapies for intestinal mucositis. The source, species, doses, treatment schedules, and mechanisms of action of polysaccharides will be discussed in detail.

## Introduction

Intestinal mucositis is a clinically significant side effect of anti-cancer therapies, characterized by ulcerative lesions along the mucous membrane lining the gastrointestinal tract (Kwon, 2016). It occurs in approximately 40% of patients undergoing standard-dose chemotherapy (Naidu et al., 2004); and 60–100% of patients receiving high doses of chemotherapy, radiation therapy, and bone marrow transplantation (Köstler et al., 2001; Logan et al., 2007). Intestinal mucositis has been associated not only with a range of significant adverse symptoms, including severe diarrhea in 5–44% of patients (Krishnamurthi and Macaron, 2021), marked weight loss, and reduced nutrient absorption, but also with limited patient’s ability to tolerate treatment, thus, delaying subsequent cycles or leading to premature discontinuation (Basile et al., 2019).

Mucositis clinical symptoms result from epithelial injury, followed by a complex series of biological events that occur in the different cellular and tissue compartments of the mucosa (Sonis, 2004). In some cases, local and systemic secondary infections are observed, increasing costs with medical care and hospitalization (Gibson et al., 2013). Thus, intestinal mucositis has a notable negative impact on patient’s clinical outcome, and its complications can even lead to death in more severe cases (Al-Dasooqi et al., 2013). Currently, there are no preventive strategies or adequate treatments for intestinal mucositis (Gibson et al., 2013). Therapy mainly focuses on attenuating mucositis symptoms (Kuiken et al., 2017). To aid the clinical management of intestinal mucositis, the Mucositis Study Group of the Multinational Association of Supportive Care in Cancer/International Society of Oral Oncology (MASCC/ISOO) developed a guideline containing updated information on therapeutic alternatives for this disorder. These include mucosal coating agents, anesthetics and analgesics, growth factors and cytokines, antimicrobials, cryotherapy, and natural agents (Elad, 2020).

In this context, polysaccharides extracted from natural products represent an interesting strategy for the development of novel therapies and interventions for intestinal mucositis. This article summarizes potential therapeutic polysaccharides or polysaccharide extracts that have been studied for the treatment of this disorder induced by conventional chemotherapy. The review discusses the main animal models used for the study of intestinal mucositis and the mechanisms involved in the protective actions of polysaccharides.

## Animal Models of Intestinal Mucositis

Gastrointestinal mucositis is a comprehensive term used to refer to the mucosal damage caused by antineoplastic treatment (i.e., chemotherapy and/or radiotherapy) (Cinausero et al., 2017). Treatment regimen can induce lesions in the oral cavity, pharynx, larynx, stomach, and intestine, with severe consequences for the morbidity and mortality of cancer patients (Sonis et al., 2015).

Rodent models of intestinal mucositis have been essential to allow the dissection of the mechanisms involved in chemotherapy-induced toxicity (Bowen et al., 2011). The different treatment schemes, including dosing schedules, routes of administration, duration of treatments, and period of experimental follow-up, generally reflect the five-phase model of intestinal mucositis: initiation, messenger signaling, signal amplification, ulceration with inflammation, and healing (Sonis et al., 2004; Sougiannis et al., 2021). The main drugs used in rodent models of intestinal mucositis include 5-fluorouracil (5-FU) and irinotecan; however, cyclophosphamide, cisplatin, and etoposide are also used (Ribeiro et al., 2016; Wardill et al., 2019).

**FIGURE 1:**
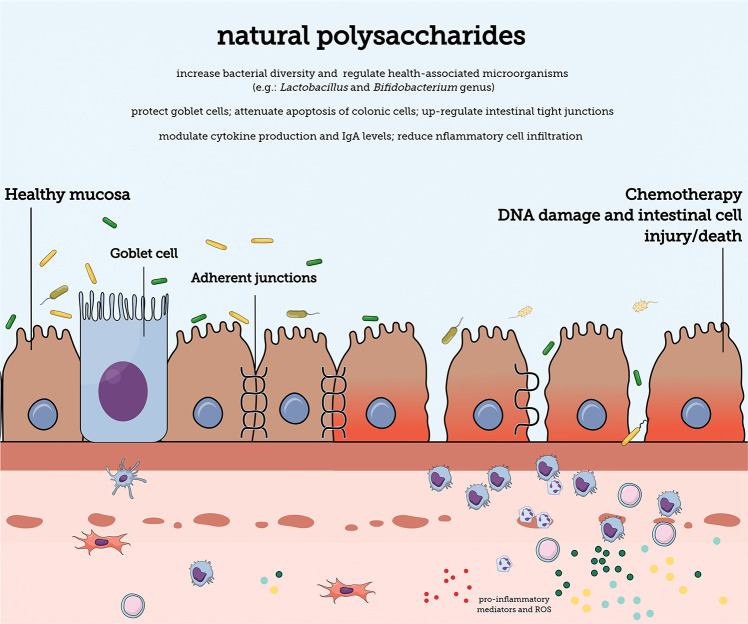
The gastrointesti nal mucosal barrier. Natural polysaccharides can contain different structural characteristics and can protect the mucosa against damage caused by chemotherapy antineoplastic treatment. The mucosal barrier contains diverse barriers (e.g., mucus layer and adherent junction proteins) that promote protection against internal and external harmful substances. A single epithelial cell layer forms the physical barrier against the entry of antigens. The lamina propria is densely populated by cells of the immune system that provide protection against damaging substances.

Although each drug previously mentioned has a specific mechanism of action, in general, they trigger mucosal injury by similar mechanisms (Figure 1). The generation of reactive oxygen species (ROS) (Bhattacharyya et al., 2014), and inflammation – characterized by the production of cytokines, chemokines, and prostaglandins, trigger direct damage to cellular components such as nucleic acids, proteins, and lipids (Sonis, 2002; Logan et al., 2007). The activation of pathways such as caspase, protein kinases, as well as an unregulated expression of metalloproteinase, contribute to the damaging process of the intestinal mucosa, leading to apoptosis of basal and submucous epithelial cells and atrophic changes (Cinausero et al., 2017). All these irregular responses contribute to the intestinal tissue’s vulnerability to ulcerations and infections (Blijlevens and Sonis, 2007; Al-Dasooqi et al., 2010).

5-Fluorouracil is commonly used for the treatment of malignant tumors. It is an analog of uracil with a fluorine atom at the C-5 position in place of hydrogen. Intracellularly, 5-FU is converted into active metabolites that disrupt RNA synthesis and the action of thymidylate synthase, thus, killing malignant cells (Longley et al., 2003). In animals, 5-FU administration (25–450 mg/kg) results in weight loss, diarrhea, and increased mortality (Araújo et al., 2015; Pereira et al., 2015). 5-FU triggers inflammation of the small intestine, characterized by the infiltration of immune cells (Chang et al., 2012). Decreased cellularity in the small intestine and increased apoptosis in both the ileum and colon are also observed by TUNEL and western blot analysis (Justino et al., 2014; Yeung et al., 2015). Similarly, irinotecan, a topoisomerase I inhibitor, has also been broadly employed to investigate the pathogenesis of mucositis (Wardill et al., 2013). By binding to the topoisomerase I-DNA complex, irinotecan prevents the rebinding of the DNA strand, interfering with the replication fork thus, inducing replication arrest and lethal double-stranded breaks in the DNA (Fujita et al., 2015). Its administration to animals (75–300 mg/kg) causes severe diarrhea, increases intestinal motility, and triggers mucosal injury (Di Paolo et al., 2006; Gibson et al., 2007), associated with histopathological alterations and increased intestinal inflammation mediated by enhanced cytokine production (Melo et al., 2008). Cyclophosphamide is also widely used for the treatment of malignancies and known to induce intestinal mucositis. It is an alkylating agent of the nitrogen mustard class and in its activated form, phosphoramide mustard, binds to the DNA interfering with its normal function. Its main cytotoxic effect is due to cross-linking of nucleic acid strands and inhibition of protein synthesis (Iqubal et al., 2018). In animals, cyclophosphamide (50–400 mg/kg) induces changes similar to those described for 5-FU, such as diarrhea and dyskinesia (Wardill et al., 2019). Some studies also observed this chemotherapy leads to increased intestinal permeability (Xiang et al., 2010) and epithelial destruction, in addition to dysfunction of the intestinal immunity (Owari et al., 2012). Cisplatin (alkylating agent) and etoposide (topoisomerase II inhibitor), either alone or in combination (Safonova et al., 2019), and radiation therapy (Bowen et al., 2011) are less commonly used but are also valuable models (Wardill et al., 2019) which provide information regarding the underlying mechanisms of cancer therapy-induced mucosal damage.

## Natural Polysaccharides

As defined by the European Nutraceutical Association (Ena) (2016), nutraceuticals are “nutritional products that provide health and medical benefits, including the prevention and treatment of diseases.” Nutraceuticals include biologically active molecules present in food such as those of extracts and herbs, as well as minerals, vitamins, carbohydrates, lipids amongst other nutrients. The global nutraceutical market reached USD 382.51 billion in 2019 and it is expected to continue growing 8.3% by 2027 (Global Market Research, 2021). Those found in the market have a range of applications from appetite suppression to immunomodulation (Sachdeva et al., 2020).

Carbohydrates are mostly found as polysaccharides – macromolecules of high molecular weight formed by polymers of monosaccharides such as pentoses and hexoses linked by glycosidic bonds. Recent studies have identified various biological activities of polysaccharides with wide structural diversity from different sources including algae (Xu et al., 2017; Manlusoc et al., 2019; Van Weelden et al., 2019), herbs (Zhang et al., 2013, 2015; Li et al., 2018a; Maria-Ferreira et al., 2018), fruits (Potterat, 2010; Ahmadi Gavlighi et al., 2018), squids (Zuo et al., 2014a, 2015a,2015b; Gu et al., 2017; Chen et al., 2020a), bacteria (Ciszek-Lenda et al., 2011; Jones et al., 2014), and mushrooms (Kothari et al., 2018; Chen et al., 2020b; Jiang et al., 2020).

Polysaccharides can be classified according to their shape, monomeric units, and electric charge (BeMiller, 2018). By shape, polysaccharides can be linear, branched, short branches on an essentially linear backbone, or branch-on-branch structures. By monomeric units, they can be either homo- or heteroglycans, and by charge, neutral or anionic. Polysaccharides are also classified according to their source: (i) starch, cellulose, and exudate gums from plants; (ii) alginates and galactans from algae; (iii) chitin, chitosan, and glycosaminoglycans from animals; (iv) dextran, pullulan and, gellan and xanthan gums from microorganisms; and (v) glucans from mushrooms (Friedman, 2016; Choong et al., 2018; Li et al., 2018b; Yadav and Karthikeyan, 2019).

Polysaccharides are non-digestible fibers (prebiotics) which may confer health benefits through different mechanisms (Figure 1). In the context of intestinal mucositis, their protective effects may be due to the following actions: (i) modulation of immune and inflammatory responses (Zhao et al., 2020), (ii) antioxidant (Wang H. et al., 2013) and (iii) antibacterial potential (Coma et al., 2015), (iv) protection of the mucosa and mucin regulation (Zuo et al., 2015a,b; Meldrum et al., 2017), and (v) alterations in the intestinal microbiota (Tang et al., 2019). The later, comprises a positive regulation of health-associated microorganisms such as those of the *Lactobacillus* and *Bifidobacterium* genus, and increased bacterial diversity (Tang et al., 2019).

The potential of different natural polysaccharides to protect against the inflammatory/oxidative damage of the intestinal mucosa and their impacts on the histopathological changes of intestinal mucositis will now be discussed.

## Effects of Natural Polysaccharides on Chemotherapy-Induced Intestinal Histopathological Changes

Animal models are reliable in mimicking the human intestinal mucositis (Wardill et al., 2019) and have been essential for the research of novel therapeutic and prevention strategies for this condition. Animals with intestinal mucositis typically show a range of gastrointestinal symptoms, including bloody diarrhea, ulcerations, and diverse structural tissue alterations (Cinausero et al., 2017). Antineoplastics such as 5-FU and cyclophosphamide induce villus atrophy, crypt apoptosis and decrease the number of intestinal cells (Pritchard et al., 1998; Logan et al., 2009; Yang et al., 2013; Cinausero et al., 2017). Mucus secretion, tight junction integrity and intestinal permeability are also altered by chemotherapy (Song et al., 2013; Yang et al., 2013; Yeung et al., 2015; Cinausero et al., 2017).

Polysaccharides isolated from different sources have been explored as potential treatments for intestinal mucositis. The identified mechanisms of action of these macromolecules in the histological alterations of mucositis are summarized in Table 1.

**TABLE 1:** *In vivo* effects of natural polysaccharides on chemotherapy-induced intestinal mucositis.

Animal model of intestinal mucositisy				Polysaccharide							References
Species	Strain	Antineoplastic chemotherapy	Antineoplastic dose	Source	Polysaccharide compound/preparation	Dose	Route and frequency of administration	Effects on tissue histology	Effects on inflammation	Effects on oxidative stress	
Mice	Male Balb/c	Cyclophosphamide	50 mg/kg	*Acaudina molpadioides*	Fucoidan or fucoidan products	50 mg/kg	Gavage, once a day, for 28 days	Reduction of intestinal mucosa injury with preservation of villus length and crypt depth	Increase of intestinal IFN-γ/IL-4 ratio, IL-6/IL-10 production, and mucosal IgA levels	**–**	Zuo et al., 2014b
		Cyclophosphamide	50 mg/kg	*Ommastrephes bartrami*	Squid ink polysaccharide extract	50–200 mg/kg	Gavage, once a day for 28 days	Up-regulation of intestinal epithelium barrier tight (occluding and ZO-1) and adherens junctions (E-cadherin)	–	–	Zuo et al., 2015a
		Cyclophosphamide	50 mg/kg	*Ommastrephes bartrami*	Squid ink polysaccharide extract	50–200 mg/kg	Gavage, once a day for 28 days	Protection of the intestinal layer due to up-regulation of cytokeratin 18 and mucin 2 mRNA by goblet cells	–	–	Zuo et al., 2015b
		5-fluorouracil	25 mg/kg	*Poria cocos*	Carboxymethyl pachyman	50–100 mg/kg	Gavage, once a day, for 14 days	Prevention of colon and villi shortening, crypt disruption, goblet cell reduction, mucosa and muscle layer thinning, attenuation of the apoptosis of colonic cells	Reduction of inflammatory cell infiltration with attenuation of the expression of NF-κB and p38 MAPK	Suppression of ROS production and increase of the levels of CAT, GSH-Px, and GSH	Wang et al., 2018
	Female C57BL/6	5-fluorouracil	50 mg/kg	Crab shell	Chitin or chitosan	2 mg/animal	Intragastric, once a day, for 10 days	Reduction of mucosal ulceration and villus height, prevention of the apoptosis of intestinal crypt cells	Attenuation of the influx of MPO-positive cells	–	Koizumi et al., 2017
	Male C57Bl/6	Cisplatin + paclitaxel and cisplatin + irinotecan	Cisplatin (2.5 mg/kg) Paclitaxel (12 mg/kg) Cisplatin + irinotecan (10 mg/kg)	*Tussilago farfara L.*	Monosaccharide composition, uronic acid content	20 mg/kg	Intraperitoneal, for 14 days	Decrease of DNA damage in intestinal cells	–	–	Safonova et al., 2019
Rats	Male Sprague-Dawley	5-fluorouracil	50 mg/kg	*Crassostrea hongkongensis*	Polysaccharide-based nutrition formula	2.0 g/kg	Gavage, once a day, for 13 days	Reduction of ulceration with diminished villus atrophy and increase of the mucosa thickness	Increase of plasma levels of IFN-γ and IL-10, and augmentation of IL-2 production	–	Cai et al., 2018
	Female Dark Agouti	5-fluorouracil	150 mg/kg	–	Fructo-, galacto-, or mannan-oligosaccharides	50 mg/kg	Gavage, once a day, for 13 days	Improvement of the intestinal integrity by galacto- oligosaccharides with no effects on mucin or in the number of goblet cells	No effects on tissue MPO-positive nor in peripheral blood cells	–	Yazbeck et al., 2019
Human	Male and female patients with colorectal cancer	Oxaliplatin, 5-fluorouracil, and folinic acid combination therapy	–	–	β-glucan	50 mg/day	Oral, for at least 7-days	–	Prevention of the chemotherapy-induced peripheral blood leukocyte and neutrophil number reduction	–	Karaca et al., 2014

*Poria cocos* is a saprophytic fungus, popularly used in traditional East-Asian medicine (Wang Y. Z. et al., 2013). Wang Y. Z. et al. (2013) modified its main constituent, pachyman, to a carboxymethylated pachyman (CMP), and tested it in the 5-FU-induced intestinal mucositis model. CMP (50–100 mg/kg) reduced colon injury in CT26 tumor-bearing male Balb/c mice treated with 5-FU. CMP also decreased the disease score and reversed intestinal shortening (Wang et al., 2018).

The protective effects of chitosan, a linear polysaccharide of the chitin family, in the digestive system were previously demonstrated (Kimura and Okuda, 1999; Kimura et al., 2001). In the 5-FU intestinal mucositis model, chitosan (2 mg/animal) prevented tissue damage, by reducing the loss of the intestinal architecture, as well as mucosal ulceration, villus height, and inflammatory cell infiltration, in female C57BL/6 mice (Koizumi et al., 2017).

In addition to the protective effects of isolated polysaccharides, immunonutrition formulas containing polysaccharides were also shown to improve the outcome of intestinal mucositis. In fact, the administration of OPNF (*Crassostrea hongkongensis* oyster polysaccharide-based nutrition formula, 2 g/kg) reduced the edema of the lamina propria and jejunum villi atrophy in Sprague-Dawley male rats receiving 5-FU chemotherapy. OPNF restored villus height, width, and mucosal thickness; and reduced the injury of the intestinal mucosa as shown by scanning electron microscopy (Cai et al., 2018). The same authors also demonstrated that OPNF inhibits tumor growth by improving leukocyte and lymphocyte indexes in S180 tumor-bearing mice undergoing therapy with 5-FU, suggesting that oyster polysaccharides may act as immunological stimulants (Cai et al., 2016).

Fucoidan is a fucose-enriched and sulfated polysaccharide, mainly sourced from seaweed which has been extensively investigated (Zhao et al., 2018) and suggested to present anticancer, antiviral, and anti-diabetic properties (Luthuli et al., 2019). The oral administration of different *Acaudina molpadioides* fucoidans (10–500 kDa) at a dose of 50 mg/kg, improved the histological morphology of the small intestine in male Balb/c mice challenged with cyclophosphamide. The length of the small intestine was restored by fucoidan. Also, the length and the villus/crypt ratio were increased, while the crypt depth was diminished in mice that received fucoidan. Interestingly, the results indicate that fucoidans of molecular weights ranging from 50 to 500 kDa present the greatest protective effects in intestinal mucositis (Zuo et al., 2014b).

Another investigated polysaccharide is a marine glycosaminoglycan isolated from the squid ink (*Ommastrephes bartrami*, OBP). It was demonstrated in two studies, that OBP (50–200 mg/kg) protects male Balb/c mice against cyclophosphamide-induced intestinal damage (Zuo et al., 2015a,b). This effect was associated with an enhanced intestinal mRNA and protein expression of myosin, occludin, E-cadherin, and zonula occludens (ZO)-1. This was especially noted in animals treated with 200 mg/kg of OBP (Zuo et al., 2015a). OBP (100–200 mg/kg) was also found to prevent weight loss and decrease diarrhea severity (Zuo et al., 2015b); an effect associated with increased mRNA expression of cytokeratin 18 and mucin 2, and larger mucin-positive areas in the intestinal villi. The same study also demonstrated that OBP enhances the quantity of goblet cells in the epithelium.

Since mushrooms and fruits are routinely present in the human diet, polysaccharides isolated from them have been extensively explored as sources of compounds in recent years. *Ganoderma atrum*-derived polysaccharide (PSG-1, 25–100 mg/kg) was assessed in specific pathogen-free female BALB/c mice challenged with cyclophosphamide. PSG-1 (50 and 100 mg/kg) enhanced the number of goblet cells. Moreover, all tested doses of PSG-1 led to an increase of intestinal claudin-1, occluding, and ZO-1 protein levels, in comparison to vehicle animals receiving cyclophosphamide. The highest tested dose of PSG-1 presented the most pronounced effects on claudin-1 and ZO-1 expressions; whilst the lowest dose presented the greatest effect on occludin levels (Ying et al., 2020).

A polysaccharide isolated from Longan (LP), a fruit derived from *Dimocarpus longan* Lour, was also studied. LP (100–400 mg/kg) was tested in the cyclophosphamide mucositis model and found to partially protect the intestinal structure by reducing damage in comparison to vehicle-treated mice with cyclophosphamide-induced mucositis. The highest tested dose of LP (400 mg/kg) enhanced mucin 2 mRNA expression. LP also upregulated the mRNA and protein expressions of claudin-1, claudin-4, and ZO-1, especially at the highest dose, without altering occludin expression. On the other hand, E-cadherin mRNA and protein expressions were increased by LP, with no dose-effect relationship (Bai et al., 2019).

Finally, Safonova et al. (2019) used a combination therapy of cisplatin and irinotecan to evaluate the protective effects of *Tussilago farfara* L. polysaccharides on the small intestinal epithelium. *T. farfara* L. polysaccharides reduced the DNA damage caused by the chemotherapy denoted by increased percentage of DNA in the tail of the comet and apoptotic DNA in the small intestinal epithelium in comparison with those of the negative control group (Safonova et al., 2019).

## Effects of Natural Polysaccharides on Intestinal Mucositis-Associated Inflammation and Oxidative Stress

Inflammation and oxidative stress account for as underlying mechanisms of the intestinal pathological changes caused by chemotherapy (Sougiannis et al., 2021). Chemotherapy triggers an initial tissue response in which DNA damage occurs, followed by an intense production of ROS, up-regulation of inflammatory genes, inflammasome activation and influx of activated immune cells. The enhanced generation of cytokines such as tumor necrosis factor-α, IL-1β, and IL-6, and chemokines amplify the accumulation of inflammatory cells in the damaged intestinal mucosa. The intense inflammation favors the occurrence of ulcerations in the intestinal epithelial layer, increasing ROS production. In parallel, the ulcers may become colonized by gut bacteria, amplifying tissue inflammation. These local changes as well as the systemic anemia and lymphocytopenia triggered by chemotherapy contribute to an inefficient healing of the intestine.

Pieces of evidence (Table 1) indicate a potential use of natural polysaccharides as therapeutic modulators of inflammation in intestinal mucositis. Indeed, apart from a single study which did not observe any benefits from prebiotics in rats with 5-FU-induced intestinal mucositis (Yazbeck et al., 2019), a variety of natural polysaccharides exhibited protective effects in different studies. Of note, these have been employed as orally administered preventive approaches to avoid intestinal inflammation and subsequent damage.

Wang et al. (2018) demonstrated the ability of *P. cocos* CMP (50–100 mg/kg) to protect the colon tissue against 5-FU-induced damage in Balb-c mice, by reducing inflammatory cell infiltration and pro-inflammatory gene expression and reducing oxidative stress. The protective effects of natural polysaccharides on chemotherapy-induced intestinal mucositis have been confirmed by different studies with other antineoplastics and animal strains including C57BL/6 and rats (Koizumi et al., 2017; Cai et al., 2018; Safonova et al., 2019), in addition to humans (Zhang et al., 2018). In animals with intestinal damage caused by 5-FU, the administration of crab shell chitin/chitosan (2 mg/animal) in C57BL/6 mice (Koizumi et al., 2017) or a polysaccharide-based nutrition formula (2 g/kg) from *C. hongkongensis* in rats impaired inflammation by reducing the influx of MPO-positive cells and increasing cytokine production (Cai et al., 2018).

Similarly, *A. molpadioides* fucoidan and its products (50 mg/kg) protected against the intestinal damage caused by cyclophosphamide in Balb-c mice by increasing intestinal IFN-γ/IL-4 ratio, IL-6/IL-10 production, and mucosal IgA levels (Zuo et al., 2014b). Another study with β-glucans in patients with colorectal cancer undergoing combination chemotherapy with oxaliplatin, 5-FU, and folinic acid, prevented drug-induced peripheral blood leukocyte and neutrophil number reduction (Karaca et al., 2014).

## Effects of Natural Polysaccharides on Human Intestinal Mucositis

For the best of our knowledge, there are few reports on the effects of polysaccharides in patients with intestinal mucositis. Oral β-glucan, for instance, was found to improve immunity in patients undergoing chemotherapy, with no evidence on its ability to protect against intestinal damage (Steimbach et al., 2020). Also, a retrospective study demonstrated that the same compound reduces diarrhea in patients suffering from colorectal cancer undergoing chemotherapy (Karaca et al., 2014); this study did not assess any other relevant aspect of intestinal mucositis (Karaca et al., 2014). None of the aforementioned studies offered sufficient information on whether polysaccharides are beneficial in human intestinal mucositis. The limited information on the effects of polysaccharides in patients undergoing chemotherapy highlights the need for high-quality trials using multiple measures to assess their impact on all side effects induced by antineoplastics. In this context, further investigations aimed at determining the unknown molecular/underlining mechanisms of polysaccharides as well as how these macromolecules affect the intestinal microbiota are of great value for the clinical management of the intestinal mucositis.

## Conclusion and Perspectives

All the non-clinical studies discussed here, indicate that regardless of the polysaccharide source or type, these, when given orally, present a great ability to prevent the intestinal damage caused by antineoplastic drugs such as 5-FU and cyclophosphamide, as well as polychemotherapy. Their effects are suggested to be due to their ability to regulate the inflammatory/immune responses and attenuate oxidative stress, resulting in enhanced integrity of the intestinal mucosa *via* regulation of mucin production and increased expression of different tight junction molecules. Despite the exciting evidence, their clinical benefits remain to be confirmed in patients with intestinal mucositis, highlighting the need for further detailed studies with these macromolecules.

## Author Contributions

KS, BC, LB, LM, and LS did the bibliographic survey and extracted data. DM-F and EF contributed to the conception and design of the manuscript and drafted and critically revised the manuscript. All authors gave final approval and agreed to be accountable for all aspects of the work.

## Conflict of Interest

The authors declare that the research was conducted in the absence of any commercial or financial relationships that could be construed as a potential conflict of interest.
